# Management and Clinical Outcome of Posterior Reversible Encephalopathy Syndrome in Pediatric Oncologic/Hematologic Diseases: A PRES Subgroup Analysis With a Large Sample Size

**DOI:** 10.3389/fped.2021.678890

**Published:** 2021-07-01

**Authors:** Marady Hun, Min Xie, Zhou She, Amin S. Abdirahman, Cuifang Li, Feifeng Wu, Senlin Luo, Phanna Han, Rithea Phorn, Pan Wu, Haiyan Luo, Keke Chen, Jidong Tian, Wuqing Wan, Chuan Wen

**Affiliations:** ^1^Division of Hematology and Tumor, Children's Medical Center, The Second Xiangya Hospital, Central South University, Changsha, China; ^2^Department of Ophthalmology, The Second Xiangya Hospital, Central South University, Changsha, China; ^3^Department of General Surgery, The Second Xiangya Hospital, Central South University, Changsha, China; ^4^Department of Hematology, Hunan Children's Hospital, University of South China, Changsha, China; ^5^Department of Hematology, Children's Medical Center, Hunan Provincial People's Hospital, Hunan Normal University, Changsha, China

**Keywords:** posterior reversible encephalopathy syndrome, chemotherapy, hematopoietic stem cell transplantation, children, oncologic/hematologic diseases, neurotoxicity, management

## Abstract

This study investigated the management and clinical outcomes along with associated factors of posterior reversible encephalopathy syndrome (PRES) in childhood hematologic/oncologic diseases. We present data from children with hematologic/oncologic diseases who developed PRES after treatment of the primary disease with chemotherapy and hematopoietic stem cell transplantation (HSCT) at 3 medical centers in Changsha, China from 2015 to 2020, and review all previously reported cases with the aim of determining whether this neurologic manifestation affects the disease prognosis. In the clinical cohort of 58 PRES patients, hypertension [pooled odds ratio (OR) = 4.941, 95% confidence interval (CI): 1.390, 17.570; *P* = 0.001] and blood transfusion (OR = 14.259, 95% CI: 3.273, 62.131; *P* = 0.001) were significantly associated with PRES. Elevated platelet (OR = 0.988, 95% CI: 0.982, 0.995; *P* < 0.001), hemoglobin (OR = 0.924, 95% CI: 0.890, 0.995; *P* < 0.001), and blood sodium (OR = 0.905, 95% CI: 0.860, 0.953; *P* < 0.001), potassium (OR = 0.599, 95% CI: 0.360, 0.995; *P* = 0.048), and magnesium (OR = 0.093, 95% CI: 0.016, 0.539; *P* = 0.008) were protective factors against PRES. Data for 440 pediatric PRES patients with hematologic/oncologic diseases in 21 articles retrieved from PubMed, Web of Science, and Embase databases and the 20 PRES patients from our study were analyzed. The median age at presentation was 7.9 years. The most common primary diagnosis was leukemia (62.3%), followed by solid tumor (7.7%) and lymphoma (7.5%). Most patients (65.0%) received chemotherapy, including non-induction (55.2%) and induction (44.8%) regimens; and 86.5% used corticosteroids before the onset of PRES. Although 21.0% of patients died during follow-up, in most cases (93.2%) this was not attributable to PRES but to severe infection (27.3%), underlying disease (26.1%), graft-vs.-host disease (14.8%), multiple organ dysfunction syndrome (8.0%), and respiratory failure (3.4%). PRES was more common with HSCT compared to chemotherapy and had a nearly 2 times higher mortality rate in patients with oncologic/hematologic diseases than in those with other types of disease. Monitoring neurologic signs and symptoms in the former group is therefore critical for ensuring good clinical outcomes following treatment of the primary malignancy.

## Introduction

Approximately 70,000 new cases of oncologic disease diagnosed annually are among adolescents and young adults (1, 2). Over the past decades, the 5-year survival rate for pediatric cancer improved from 58% in the period from 1975 to 1977 to 83% from 2005 to 2015 and 84% from 2010 to 2016 (3–7). Acute lymphoblastic leukemia (ALL) is the most common childhood malignancy—accounting for 20% of all cancers occurring before 20 years of age (8, 9)—and has good prognosis: the current 5-year overall survival rate of childhood ALL is 90% (8). This is mainly due to the reduction of risk and adverse reactions associated with cytotoxic therapies including hematopoietic stem cell transplantation (HSCT) and chemotherapy. Posterior reversible encephalopathy syndrome (PRES), a severe neurologic complication and adverse reaction in pediatric oncologic/hematologic patients following chemotherapy and HSCT treatment (10–16), is a clinical syndrome characterized by headache, seizures, mental and visual impairment, and vomiting accompanied by reversible vasogenic edema observed by magnetic resonance imaging (MRI) that impacts the subcortical white matter of supratentorial lobes, especially in the parieto-occipital lobes (17–19).

PRES was first described in 1996 in adults with various primary diagnoses (20), and occurs less frequently in children (21). Nephrotic syndrome is a major primary cause of PRES in children (21–23). However, PRES has recently been reported in single- or multi-center studies of pediatric oncologic/hematologic diseases such as leukemia, lymphoma, solid tumors, and non-malignant disease after chemotherapy and HSCT, with high morbidity and mortality rates ranging from 2.4 to 22.6% (11, 12, 15, 18, 22, 24–28). In patients with oncologic/hematologic diseases, the main causes of death were underlying diseases, severe infection, multiple organ dysfunction syndrome (MODS), respiratory failure, graft-vs.-host disease (GVHD), and severe organ toxicity (15, 18, 25–32); and several studies found that the deaths were directly attributable to PRES (12, 18, 24, 28, 30).

Despite these recent findings, most studies to date on PRES have had small sample sizes and are case reports or series; thus, a comprehensive view of PRES in a large sample is lacking. To address this issue, in this study we investigated the features, management, and clinical outcomes of PRES in a large sample of pediatric patients with oncologic/hematologic diseases with the aim of determining whether this neurologic manifestation affects the prognosis of the primary disease.

## Methods

### Search Strategy, Selection Criteria, Quality Assessment, and Data Extraction

The multicenter cohort comprised pediatric patients treated between January 2015 and December 2020 at The Second Xiangya Hospital, Hunan Children's Hospital, and Hunan Provincial People's Hospital (all in Changsha, China). We used a retrospective matched case–control study design to analyze data for patients who developed PRES—which was diagnosed according to established clinical and neuroimaging criteria (17, 33)—after HSCT or chemotherapy for oncologic/hematologic diseases and non-HSCT chemotherapy for non-oncologic/hematologic diseases. PRES was suspected when patients experienced abrupt onset of 1 of the following symptoms: headache, seizures, visual disturbances, confusion, and radiologic findings (focal regions of brain vasogenic edema). We analyzed clinical symptoms, laboratory parameters, neuroimaging findings, treatment strategies, and outcomes from the time of diagnosis of oncologic/hematologic and non-oncologic/hematologic diseases to the onset of PRES (Tables 1, 2).

**Table 1:** Clinical and epidemiologic characteristics of patients with PRES in oncologic/hematologic diseases or non-oncologic/hematologic diseases.

**Characteristic**	**All (*n* = 58)**	**PRES in non-hematologic/oncologic diseases (*n* = 38)**	**PRES in hematologic/oncologic diseases (*n* = 20)**	**t/Z/χ^**2**^**	***P*-value**
Median age (range), years	12.00 (6.88, 28.25)	18.50 (10.80, 33.25)	6.80 (5.18, 9.10)	−4.753	0.001
Sex					
Male	32 (55.2)	19 (50.0)	13 (65.0)	1.192	0.275
Female	26 (44.8)	19 (50.0)	7 (35.0)		
Symptom/sign					
Hypertension	33 (56.9)	17 (44.7)	16 (80.0)	6.644	0.010
Fever	14 (24.1)	9 (23.7)	5 (25.0)	0.012	0.911
Suspected sepsis	12 (20.7)	2 (5.3)	10 (50.0)	15.982	0.001
Seizures	37 (63.8)	20 (52.6)	17 (85.0)	5.944	0.015
Mental impairment	31 (53.4)	19 (50.0)	12 (60.0)	0.527	0.468
Headache	26 (44.8)	23 (60.5)	3 (15.0)	10.981	0.001
Visual impairment	22 (37.9)	10 (26.3)	12 (60.0)	6.315	0.022
Vomiting	21 (36.2)	12 (31.6)	9 (45.0)	1.022	0.312
Blood transfusion (1 week before onset)	14 (24.1)	3 (7.9)	11 (55.0)	15.878	0.001
PRES-related examination					
Blood platelet count, ×10^9^/l^†^	192.50 (67.75, 318.75)	247.50 (143.75, 357.00)	39.00 (16.25, 152.25)	−4.139	0.001
Hemoglobin, g/l^‡^	106.50 (73.75, 123.25)	117.50 (104.00, 129.00)	68.50 (61.00, 90.00)	−5.081	0.001
Blood sodium before onset, mmol/l	135.20 (114.78, 140.68)	138.70 (134.90, 142.25)	112.85 (106.55, 117.85)	−5.890	0.001
Blood sodium after onset, mmol/l	136.55 (0.00, 140.18)	134.25 (0.00, 139.58)	137.95 (133.93, 140.96)	−1.913	0.056
Blood potassium, mmol/l	3.65 (3.09, 4.10)	3.90 (3.47, 4.20)	3.22 (2.66, 3.56)	−3.567	0.001
Blood magnesium, mmol/l	0.65 (0.42, 0.90)	0.84 (0.58, 0.96)	0.49 (0.41, 0.72)	−2.864	0.004
Blood calcium, mmol/l	2.09 (1.87, 2.23)	2.11 (1.93, 2.24)	1.93 (1.79, 2.20)	−1.417	0.157
CK-MB, IU/l	16.10 (4.50, 22.63)	16.85 (8.03, 24.45)	13.00 (1.50, 21.60)	−1.293	0.196
ALB, g/l	31.20 (22.25, 37.68)	33.15 (22.25, 38.58)	30.05 (6.40, 36.38)	−0.934	0.350
ALT, U/l	17.65 (8.88, 31.68)	13.00 (9.35, 21.43)	35.75 (5.55, 65.55)	−2.210	0.027
AST, U/l	22.10 (15.38, 30.20)	19.85 (14.80, 26.18)	24.60 (16.23, 51.03)	−1.556	0.120
UA, μmol/l	253.45 (97.38, 377.48)	332.55 (242.13, 420.70)	98.00 (8.20, 134.38)	−4.404	0.001
Cr, μmol/l	50.55 (17.33, 67.60)	61.50 (47.70, 90.45)	17.15 (3.00, 26.70)	−4.560	0.001
Glu, μmol/l	5.20 (1.96, 7.46)	5.60 (4.48, 7.60)	3.95 (0.00, 7.29)	−1.450	0.147
BP before onset, mmHg					
SBP	112.79 ± 15.93	122.08 ± 17.89	106.75 ± 11.30	−3.026	0.005
DBP	71.09 ± 17.95	83.31 ± 20.00	63.15 ± 11.02	−3.737	0.001
MAP	84.99 ± 16.55	96.23 ± 18.85	77.68 ± 9.71	−3.725	0.001
BP after onset, mmHg					
SBP	148.14 ± 32.71	153.55 ± 35.12	137.85 ± 25.25	−1.770	0.082
DBP	94.59 ± 25.56	97.29 ± 26.20	89.45 ± 24.10	−1.113	0.271
MAP	112.44 ± 26.94	116.04 ± 28.25	105.58 ± 23.40	−1.418	0.162
BP >140/90 mmHg	44 (75.9)	30 (78.9)	14 (70.0)	0.573	0.449
MRI lesion sites (typical)					
Bilateral	50 (86.2)	33 (86.8)	17 (85.0)	0.037	0.847
Unilateral	8 (13.8)	5 (13.2)	3 (15.0)	0.037	0.847
White matter	15 (25.9)	9 (23.7)	5 (30.0)	0.273	0.602
Occipital lobe	28 (48.3)	16 (42.1)	12 (60.0)	1.680	0.195
Parietal lobe	35 (60.3)	21 (55.3)	14 (70.0)	1.189	0.275
Frontal lobe	23 (39.7)	13 (34.2)	10 (50.0)	1.365	0.243
Temporal lobe	7 (12.1)	4 (10.5)	3 (15.0)	0.247	0.619
Other sites (Hi, Cb, Cc, Bg, Th)	24 (41.4)	20 (52.6)	4 (20.0)	5.752	0.025
MRI features					
T1+T2	46 (79.30)	30 (78.9)	16 (80.0)	0.009	0.925
Flair	26 (44.8)	18 (47.4)	8 (40.0)	0.288	0.592
DWI	28 (48.3)	21 (55.3)	7 (35.0)	2.155	0.142
ADC	11 (19.0)	8 (21.1)	3 (15.0)	0.312	0.576
MRA+MRV	2 (3.4)	2 (5.4)	0 (0.0)	1.090	0.296
Treatment					
Diuretics	42 (72.4)	23 (60.5)	19 (95.0)	7.796	0.005
Benzodiazepines/levetiracetam	35 (60.3)	18 (47.4)	17 (85.0)	7.754	0.005
Other (PHT/PB/VPA)	20 (34.5)	12 (31.6)	8 (40.0)	0.411	0.521
Antihypertensive	43 (74.1)	29 (76.3)	14 (70.0)	0.273	0.602
Outcome					
Length of stay, days	25.00 (14.00, 36.25)	19.00 (10.75, 28.75)	35.50 (26.75, 59.75)	−3.363	0.001
ICU length of stay, days	9.50 (0.00, 101.50)	0.00 (0.00, 28.50)	55.50 (20.25, 169.00)	−3.847	0.001
Non-ICU length of stay, days	2.16 (0.00, 5.63)	0.00 (0.00, 5.25)	3.25 (0.40, 7.00)	−1.211	0.226
Length of treatment, days^§^	21.00 (10.75, 33.00)	14.00 (9.00, 25.10)	34.20 (25.78, 50.35)	−3.805	0.001
Death	4 (6.9)	2 (7.1)	2 (6.7)	0.005	0.943

*Data are shown as n (%), median (upper quartile, lower quartile), or mean ± standard deviation unless indicated otherwise*.

† *Samples were collected within 5 days before diagnosis of PRES; if patients had more than 2 transfusions, the lowest blood platelet count was used for analysis*.

‡ *Samples were collected within 5 days before diagnosis of PRES; if patients had more than 2 transfusions, the lowest hemoglobin count was used for analysis*.

§ *Treatments included chemotherapy, hematopoietic stem cell transplantation, steroid, or immunosuppressant; the length of treatment was calculated from the start of treatment to the time of PRES diagnosis*.

*ADC, analog-to-digital converter; ALB, albumin; ALT, alanine aminotransferase; AST, aspartate aminotransferase; Bg, basal ganglia; BP, blood pressure; Cb, cerebellum; Cc, corpus callosum; CK-MB, creatine kinase cardiac-type isoenzyme; Cr, creatinine; DBP, diastolic blood pressure; DWI, diffusion-weighted imaging; Glu, glucose; Hi, hippocampus; ICU, intensive care unit; MAP, mean arterial pressure; MRA, magnetic resonance angiography; MRI, magnetic resonance imaging; MRV, magnetic resonance venogram; PB, phenobarbital; PHT, phenytoin; PRES, posterior reversible encephalopathy syndrome; SBP, systolic blood pressure; Th, thalamus; UA, uric acid; VPA, valproic acid*.

**Table 2:** Multivariate competing risk regression analysis.

**Variable**	**Odds ratio**	**95% CI**	***P*-value^†^**
Hypertension (yes vs. no)	4.941	1.390–17.570	0.001
Blood transfusion (yes vs. no)	14.259	3.273–62.131	0.001
Platelet count (high vs. low)	0.988	0.982–0.995	*P* < 0.001
Hemoglobin (high vs. low)	0.924	0.890–0.959	*P* < 0.001
Sodium (high vs. low)	0.905	0.860–0.953	*P* < 0.001
Potassium (high vs. low)	0.599	0.360–0.995	0.048
Magnesium (high vs. low)	0.093	0.016–0.539	0.008

† *Model likelihood ratio χ^2^ = 21.527, P < 0.05*.

*CI, confidence interval*.

**Figure 1:**
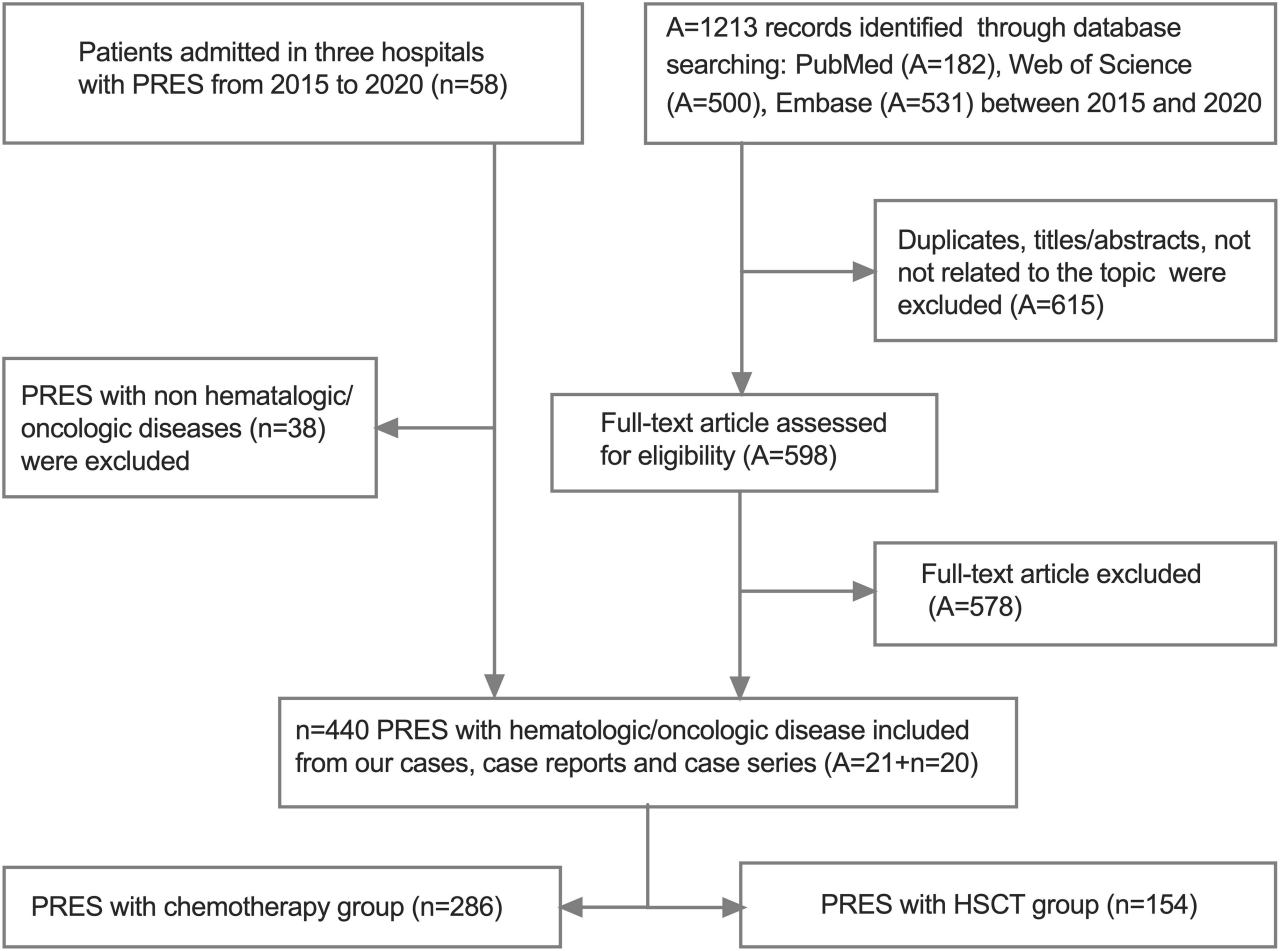
Flowchart of studies selection.

For the systematic review of previously reported cases of PRES, we searched the PubMed, Web of Science, and Embase databases for articles published in English using the following terms: (posterior leukoencephalopathy syndrome OR posterior reversible encephalopathy syndrome OR reversible posterior leukoencephalopathy syndrome OR PRES OR RPLS) AND (child OR children OR childhood). We also included 1 of the following terms to identify case reports and series of children (range 0–18 years of age) with oncologic/hematologic disease and PRES: oncologic/hematologic disease, HSCT, or chemotherapy. We manually searched the reference list of each article and selected all relevant publications from 2015 to 2020 (Supplementary Table 1). Two investigators (M. Hun and M. Xie) independently reviewed the titles and abstracts of the articles for related publications; any discrepancies were resolved by a third investigator (C. Wen). The inclusion criteria for the studies were as follows: (1) case reports, case series, or retrospective studies providing sufficient data on pediatric patients (<20 years of age) with oncologic/hematologic diseases and PRES; (2) studies estimating the relationships between PRES-related factors including primary oncologic/hematologic diseases, clinical etiology, symptoms, imaging findings, and clinical outcome in children; (3) published in English; and (4) used a self-designed table to extract data from all included literature including last name of the first author, year of publication, country, sample size, age, sex ratio, primary diagnosis, oncologic treatment at PRES onset, PRES related to treatment (anti-epileptic+anti-hypertensive), electroencephalogram (EEG) findings, symptoms/signs, neuroimaging data related to initial lesion sites, follow-up findings (follow-up times and outcome), and clinical outcome (Figure 1). Duplicated publications and studies with incomplete data, unclear outcomes, or on non-pediatric PRES were excluded.

### Statistical Analysis

Data for the multicenter cohort were analyzed using SAS v9.4 software (SAS Institute, Cary, NC, USA). Quantitative data conforming to a normal distribution are described as means and standard deviations and were analyzed with the independent-samples *t*-test. For non-normally distributed data, the median with upper and lower quartiles are presented and the non-parametric test was used to evaluate differences between groups. With the occurrence of oncologic/hematologic diseases as the dependent variable, statistically significant variables with physiologic and biochemical significance in the single-factor analysis were entered into the logistic regression model with a stepwise screening method (forward selection with entry standard = 0.05 and elimination standard = 0.10). Odds ratio (OR) was used as the risk assessment parameter. All tests were 2-sided and *P* < 0.05 was considered statistically significant.

For the systematic review, statistical analyses were performed using Excel v16.43.1 (Microsoft, Redmond, WA, USA) and SAS v9.4. Continuous variables are presented as mean ± standard deviation and categorical variables are reported as numbers and percentages in the comparison of PRES related to chemotherapy vs. HSCT in pediatric oncologic/hematologic diseases.

For the meta-analysis, we used Review Manager v5.4.1 (http://www.cochrane.org) software for statistical analyses of the included data. Between-group differences with a *P* < 0.05 were considered statistically significant, and forest plots were generated to display related factors. The quality of included studies was evaluated based on Newcastle–Ottawa Scale (NOS) score; the full score is 9 stars, and scores of 1–3, 4–6, and 7–9 stars represent low-, medium-, and high-quality studies, respectively (34).

## Results

### Incidence, Characteristics, and Clinical Features of Patients With Oncologic/Hematologic Diseases and PRES

The multicenter cohort comprised 58 pediatric PRES patients; 7 with oncologic/hematologic diseases and 38 (including 18 adults) with non-oncologic/hematologic diseases were enrolled at The Second Xiangya Hospital; and 8 and 5 PRES patients with oncologic/hematologic diseases were enrolled at Hunan Children's Hospital and Hunan Provincial People's Hospital, respectively, between 2015 and 2020. The 58 PRES patients were classified into oncologic/hematologic disease (*n* = 20) and non-oncologic/hematologic disease (*n* = 38) groups; baseline characteristics are shown in Table 1. Compared to PRES patients with non-oncologic/hematologic diseases, those with oncologic/hematologic diseases had higher rates of hypertension (*P* = 0.01), suspected sepsis (*P* = 0.001), seizures (*P* = 0.015), headaches (0.001), and blood transfusions (*P* = 0.01); higher blood platelet count (*P* = 0.001), hemoglobin (*P* = 0.001), blood sodium level at disease onset (*P* = 0.001), blood potassium (0.001), and blood magnesium (*P* = 0.004); lower systolic blood pressure (*P* = 0.005), diastolic blood pressure (*P* = 0.001), and mean arterial blood pressure (*P* = 0.001) before disease onset; more frequently used diuretics (*P* = 0.005) and benzodiazepines/levetiracetam (*P* = 0.005); had longer stays at the hospital (*P* = 0.001) and intensive care unit (*P* = 0.001); and had a longer latency from the initiation of treatment (chemotherapy/HSCT/steroid/immunosuppressive) to PRES diagnosis (*P* = 0.001) (Tables 1, 2).

The univariate analysis showed that hypertension and blood transfusion were significantly associated with the development of PRES in both groups. Variables with significant associations (*P* ≤ 0.05) were included in the competing risk regression analysis. In the final model, the pooled OR was 4.941 (95% CI: 1.390, 17.570; *P* = 0.001) for hypertension and 14.259 (95% CI: 3.273, 62.131; *P* = 0.001), for blood transfusion. Protective factors against PRES were elevated platelet count (OR = 0.988, 95% CI: 0.982, 0.995; *P* < 0.001), hemoglobin count (OR = 0.924, 95% CI: 0.890, 0.995; *P* < 0.001), blood sodium (OR = 0.905, 95% CI: 0.860, 0.953; *P* < 0.001), blood potassium (OR = 0.599, 95% CI: 0.360, 0.995; *P* = 0.048), and blood magnesium (OR = 0.093, 95% CI: 0.016, 0.539; *P* = 0.008) (Table 2).

**Figure 2:**
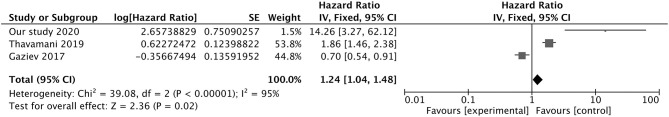
Forest plot for incidence of transfusion.

We carried out a meta-analysis of 3 studies including the present investigation and studies by Thavamani et al. (NOS score = 9) (22) and Gaziev et al. (NOS score = 7) (15) to determine whether blood transfusion is a risk factor for PRES. The results of the heterogeneity test [χ^2^ = 39.08, df = 2, *I*^2^ = 95%, *P* = 0.00001 (*Q*-test)] indicated low homogeneity between the 3 studies according to Cochrane criteria (35). We examined the funnel plot for asymmetry but found that it was within the acceptable range (Supplementary Figure 1). The mixed-effect or pooled hazard ratio of the 3 studies (1.24, 95% CI: 1.04, 1.48) was significant (*Z* = 2.36, *P* = 0.02), indicating that blood transfusion had a significant effect on the occurrence of PRES in pediatric oncologic/hematologic diseases (Figure 2).

### Systematic Review of Studies of Patients With PRES and Oncologic/Hematologic Diseases

The review of the literature ultimately yielded 21 PRES articles (11, 12, 15, 18, 24–26, 28, 31, 36–46) comprising a total of 440 pediatric PRES patients with hematologic/oncologic diseases, which were included in the meta-analysis along with the data of the 20 PRES patients from the present study (Supplementary Table 1 and Figure 1).

The median age at PRES presentation was ~7.9 years; 93 patients (38.1%) were between 10 and 19 years old, and 237 (56.6%) were male (Table 3). The most common primary diagnosis was leukemia (62.3%), followed by solid tumors (7.7%) and lymphoma (7.5%); 22.5% of patients had non-malignant disease. Chemotherapy was the most common treatment (65.0%) and the majority of patients (55.2%) were treated with a non-induction regimen, with an induction regimen used in 44.8% of cases. Additionally, 86.5% of patients used corticosteroids before the onset of PRES. Benzodiazepam (54.9%), diazepam (47.1%), and levetiracetam (47.1%) were the commonly used anti-epileptics, and 74% of patients used anti-hypertensive agents. In terms of imaging features, abnormal signals were observed in the following brain regions: occipital cortex (73.8%), parietal cortex (58.0%), frontal cortex (38.7%), and temporal cortex (26.5%). In the follow-up, there was complete resolution in 76.1% of cases (≤30 days, 83.3% vs. >30 days, 70.7%), partial resolution in 7.0% (≤30 days, 5.6% vs. >30 days, 12.2%), and residual lesions in 16.9% (≤30 days, 11.1% vs. >30 days, 17.1%). EEG revealed focal slowing in 66.0%, diffuse slowing in 37.9%, and periodic lateralized epileptiform discharge in 7.4%, with abnormal activity in the occipital (66.7%), temporal (22.2%), parietal (16.7%), and frontal (1.8%) cortices. A total of 88 patients (21.0%) died during follow-up; however, most of the deaths (93.2%) were not attributable to PRES but to severe infection (27.3%), underlying disease (26.1%), GVHD (14.8%), MODS (8.0%), and respiratory failure (3.4%), with only 6 deaths (6.8%) resulting from PRES. The cause of death was unknown or multifactorial in 13.6% of cases (Table 3).

**Table 3:** Demographic and clinical characteristics and outcomes of PRES patients with pediatric oncologic/hematologic diseases.

**Characteristic**	**Number of patients (%)**
Median age, years	7.9
Age groups, years	
0.5–9	151 (61.9%)
10–19	93 (38.1%)
Missing	196 (44.5%)
Sex	
Male	237 (56.6%)
Female	182 (43.4%)
Missing	21 (5.0%)
Primary diagnosis	
Leukemia	274 (62.3%)
Lymphoma	33 (7.5%)
Solid tumor	34 (7.7%)
Non-malignant disease	99 (22.5%)
Oncologic treatment	
Chemotherapy	286 (65.0%)
Hematopoietic stem cell transplantation	154 (35.0%)
Induction	128 (44.8%)
Non-induction	158 (55.2%)
Steroid (Pred/MP)	128 (86.5%)
Median time to PICU admission, days^†^	5.76 days
PRES treatment	
Benzodiazepam	28 (54.9%)
Diazepam/clobazam/lorazepam	24/3/1
Levetiracetam	24 (47.1%)
Other	25 (49.0%)
Mid/PHT/PB/VPA	10/6/5/4
Anti-hypertensive	47 (74.6%)
Diu/ACE-I/CCB/ARB/other	23/18/15/13/15
Diagnostic test	
MRI findings (all performed)	279
Occipital lobe	206 (73.8%)
Parietal lobe	162 (58.0%)
Frontal lobe	108 (38.7%)
Temporal lobe	74 (26.5%)
All/(missing/positive/NA/normal)	161/(123/29/6/3)
CT findings available^†^	18
Normal/positive	3/15
EEG findings (all performed)	132/370
All positive	116 (87.9%)
Normal	16 (12.1%)
Focal slowing	87 (66.0%)
Diffuse slowing	50 (37.9%)
PLEPD	4 (7.4%)
Occipital lobe	36 (66.7%)
Temporal lobe	12 (22.2%)
Parietal lobe	9 (16.7%)
Frontal lobe	1 (1.8%)
Last MRI follow-up available lesion site^†^	71/130 (12 follow-up time NA)
Complete resolution	54 (76.1%)
Partial resolution	5 (7.0%)
Residual lesion	12 (16.9%)
Last MRI follow-up at ≤30 days^†^	18/59
Complete resolution	15 (83.3%)
Partial resolution	1 (5.6%)
Residual lesion	2 (11.1%)
Last MRI follow-up at >30 days^†^	41 (69.5%)
Complete resolution	29 (70.7%)
Partial resolution	5 (12.2%)
Residual lesion	7 (17.1%)
Outcome	
Alive	330/418 (79.0%) (3 to lost follow-up, 19 NA)
Dead	88 (21.0%)
Chemotherapy	22 (5.3%)
HSCT	39 (9.3%)
Chemotherapy+HSCT	27 (6.5%)
Leukemia	14 (10.1%)
Lymphoma	7 (21.2%)
Solid tumor	4 (12.0%)
Non-malignant disease	13 (13.1%)
Relapse	5 (83.3%)
Cause of death	
Underlying disease	23 (26.1%)
Infection	24 (27.3%)
Multiple organ dysfunction syndrome	7 (8.0%)
Respiratory failure	3 (3.4%)
GVHD	13 (14.8%)
Complication	12 (13.6%)
PRES	6 (6.8%)

† *Most of the included data are from the multicenter cohort in this study*.

*ACE-I, angiotensin-converting enzyme inhibitor; ARB, angiotensin receptor blocker; CCB, calcium channel blocker; CT, computed tomography; Diu, diuretic; EEG, electroencephalogram; GVHD, graft-vs.-host disease; HSCT, hematopoietic stem cell transplantation; Mid, midazolam; MP, methylprednisolone; MRI, magnetic resonance imaging; NA, not available; PB, phenobarbital; PHT, phenytoin; PICU, pediatric intensive care unit; PLEPD, periodic lateralized epileptiform discharge; Pred, prednisone; PRES, posterior reversible encephalopathy syndrome; VPA, valproic acid*.

### Comparison of Patients With PRES and Oncologic/Hematologic Diseases Treated With Chemotherapy vs. HSCT

The median age at presentation of PRES in patients with oncologic/hematologic diseases was 5.7 years for those treated with chemotherapy and 8.9 years for those treated by HSCT (Table 4). The demographic profile of the chemotherapy and HSCT treatment groups also differed: 27.2 and 40.3%, respectively, were between 10 and 19 years, and 57.0 and 64.5%, respectively, were male. Of the 274 patients with leukemia (62.3%), 78.0% received chemotherapy and 33.1% underwent HSCT. Among the 33 lymphoma patients (7.5%), 32 (11.2%) received chemotherapy and 1 (0.7%) was treated by HSCT; the proportions were 4.5 and 13.6%, respectively, for the 34 solid tumor patients (7.7%) and 6.3 and 52.6%, respectively, for the 99 patients with non-malignant diseases (22.5%).

**Table 4:** Comparison of demographic and clinical characteristics and outcomes of PRES patients with pediatric oncologic/hematologic diseases treated with chemotherapy vs. HSCT.

**Patients**	**All available (*n* = 440)**	**Chemotherapy (*n* = 286)**	**HSCT (*n* = 154)**
Age, years			
Median	~7.9	~5.7	~8.9
0.5–9	132 (68.0%)	89 (45.8%)	43 (59.7%)
10–19	62 (32.0%)	33 (27.2%)	29 (40.3%)
Missing	246 (55.9%)	164 (57.3%)	82 (53.2%)
Sex			
Male	129 (60.3%)	69 (57.0%)	60 (64.5%)
Female	85 (39.7%)	52 (43.0%)	33 (35.5%)
Missing	226 (51.4%)	165 (57.7%)	61 (39.6%)
Primary diagnosis			
Leukemia	274 (62.3%)	223 (78.0%)	51 (33.1%)
Lymphoma	33 (7.5%)	32 (11.2%)	1 (0.7%)
Solid tumor	34 (7.7%)	13 (4.5%)	21 (13.6%)
Non-malignant disease	99 (22.5%)	18 (6.3%)	81 (52.6%)
Symptom/sign			
Hypertension	136 (85.0%)	70 (51.0%)	66 (75.0%)
Seizures	173 (88.7%)	93 (61.0%)	80 (61.1%)
Mental impairment	109 (80.7%)	60 (83.1%)	49 (60.0%)
Headache	75 (55.4%)	26 (34.7%)	49 (59.0%)
Visual impairment	63 (32.3%)	35 (29.5%)	28 (35.2%)
Vomiting	49 (30.6%)	22 (28.4%)	27 (31.3%)
MRI typical lesion sites			
Occipital lobe	206 (73.8%)	65 (55.1%)	50 (62.5%)
Parietal lobe	162 (58.0%)	72 (61.0%)	37 (46.3%)
Frontal lobe	108 (38.7%)	54 (45.8%)	31 (38.8%)
Temporal lobe	74 (26.5%)	26 (22.0%)	26 (32.5%)
Missing	161 (36.6%)	168 (58.7%)	74 (48.1%)
PRES treatment^†^			
Benzodiazepines	28 (54.9%)	23 (82.1%)	5 (17.9%)
Levetiracetam	24 (47.1%)	22 (91.7%)	2 (8.3%)
Other (Mid/PHT/PB/VPA)	25 (49.0%)	19 (76.0%)	6 (24.0%)
Outcome			
Alive	330 (79.0%)	211 (74.0%)	119 (77.3%)
All dead	88 (21.0%)	NA	NA
Dead	61 (14.5%)	22 (7.7%)	39 (25.3%)
Dead (chemotherapy+HSCT)	27 (6.5%)	NA	NA
Missing	22 (5.0%)	NA	NA

† *Most of the included data are from the multicenter cohort in this study*.

*HSCT, hematopoietic stem cell transplantation; Mid, midazolam; MRI, magnetic resonance imaging; PB, phenobarbital; PHT, phenytoin; PRES, posterior reversible encephalopathy syndrome; VPA, valproic acid*.

**Figure 3:**
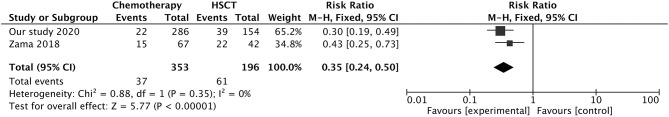
Forest plot for the comparison of mortality between Chemotherapy and HSCT.

The incidence of hypertension was higher after HSCT than after chemotherapy (66/88, 75.0% vs. 70/139, 51.0%). The rate of seizures was similar between patients treated with chemotherapy and those treated by HSCT (61.0 vs. 61.1%); meanwhile, the chemotherapy group had higher rates of mental impairment (83.1 vs. 60.0%) and headache (34.7 vs. 59.0%) and lower rates of visual impairment (29.5 vs. 35.2%) and vomiting (28.4 vs. 31.3%) than the HSCT group. Significant differences were also observed by MRI between the chemotherapy and HSCT groups in terms of the affected brain areas including the occipital (55.1 vs. 62.5%), parietal (61.0 vs. 46.3%), frontal (45.8 vs. 38.8%), and temporal (22.0 vs. 32.5%) lobes. The mortality due to PRES was higher for patients treated by HSCT than in those receiving chemotherapy (25.3 vs. 7.7%). Comparison of our data with those from a previous study (12) (NOS score = 7) confirmed that chemotherapy was safer than HSCT for the treatment of pediatric oncologic/hematologic diseases with PRES (mixed-effect or pooled risk ratio = 0.35, 95% CI: 0.24, 0.50, *Z* = 5.77, *P* = 0.00001) (Figure 3), with no obvious heterogeneity between the 2 studies [χ^2^ = 0.88, df = 1, *I*^2^ = 0%, *P* = 0.35 (*Q*-test)].

## Discussion

Our retrospective analysis of the multicenter cohort of pediatric patients with PRES with oncologic/hematologic diseases revealed significant differences compared to those with non-oncologic/hematologic diseases. Because of the small number of pediatric PRES cases, some adults were included in the latter group.

**Figure 4:**
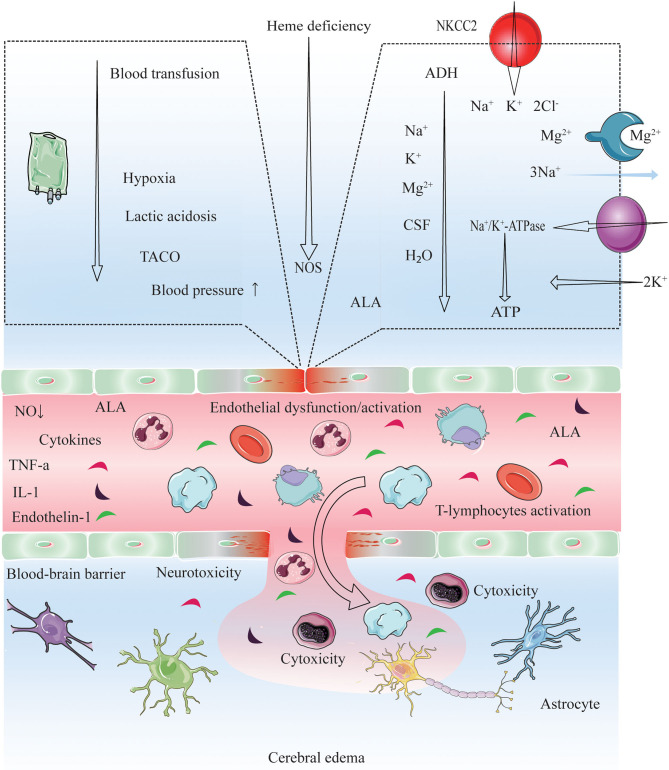
Proposed pathogenic model for cerebral edema and CNS dysfunction after conducting chemotherapy, HSCT and immunosuppressive agents. Endothelial wall inflammation disrupts the tight junctions and increase the permeability of the BBB due to high levels of circulating cytokines (TNF-α, IL-1, endothelin-1) and activating leucocytes (autoreactive T-cells). Consequently, enhanced fluid and cell diapedesis, and interstitial edema formation ensues. PRES manifestation and the dysfunction of microvasculature may be driven by the presence of checkpoint inhibitors (HSCT, chemotherapy, and immunosuppressive agent), by interactions with autoantibodies and autoreactive T-cells, and *via* abnormal secretion of angiogenic growth factors (VEGF) and proangiogenic cytokines (IL-8) (33, 66, 67), VEGF expression is increased, leading to increased vascular permeability and interstitial cerebral edema (33). Blood transfusion triggers a rapid increase in the hemoglobin, platelet, and viscosity levels, which is thought to trigger transfusion-associated circulatory overload (TACO) (68–71). Elevated blood pressure, acute hypoxia, anemia, and lactic acidosis are all risk factors for TACO (69, 72); on the other hand, acute hypoxia may decrease cerebrospinal fluid (CSF) volume, increase cerebral blood volume (CBV), and increase brain parenchyma perfusion as an early responses to hypoxia (within 40 min) (73, 74). This increase could induce acute vascular endothelium dysfunction and an elevation of vascular resistance, leading to extravasation of macromolecules into the brain. Also, the velocity of brain blood flow is shown to increase after transfusion (70, 75). Cytokines induce the expression of adhesion molecules (ICAM-1, VCAM-1), which interact with leukocytes and potentiate ROS production. ROS and ALA might cause direct endothelial cell injuries, increasing the expression of VEGF and vascular permeability. A low ATP supply impairs energy-dependent processes, such as NA^+^/K^+^ ATPase function. While an ADH excess causes ALA neurotoxicity and the effect of IL-6 in the hypothalamus might lead to an increment in ADH secretion. ADH inhibits NA^+^/K^+^ ATPase and induces NKCC2 and AQP4 in astrocytes, leading to increase ion/water influx and swelling (76). ADH excess may also lead to electrolyte disorders (hyponatremia, hypocalcemia, hypomagnesemia) (22, 63, 77–79). NO deficiency: PTX3, heme deficiency and ROS might impair NOS function, thus decreasing NO synthesis and causing endothelial dysfunction. PEPT2 dysfunction: The PEPT2*2 variant has a lower affinity for ALA than PEPT2*1, which might cause a diminished ALA efflux in the choroid plexus and a more significant ALA neurotoxicity in the brain (80). Electrolyte disorders (hyponatremia, hypocalcemia, hypomagnesemia), low CSF (81), and lack of ATP might also reduce PEPT2 function. These cascades lead to vasogenic cerebral edema, and certain precipitants are probably necessary to cause PRES and CNS dysfunction. PRES, posterior reversible encephalopathy syndrome; TACO, transfusion-associated circulatory overload; ADH, Antidiuretic hormone; ALA, 5-Aminolevulinic acid; ALAS1, 5-Aminolevulinic acid synthase-1; AQP4, Aquaporin-4; BBB, Blood-brain barrier; ICAM1, Intracellular adhesion molecule-1; VCAM-1, vascular cell adhesion molecule 1; IL, Interleukin; NKCC1, Na^+^ K^+^ 2Cl^−^ Cotransporter 1; NO, Nitric oxide; NOS, Nitric oxide synthase; PEPT2, Peptide transporter-2; PTX3, Pentraxin-3; ROS, Reactive oxygen species; TCA, Tricarboxylic acid cycle; TNF-α,Tumor necrosis factor-α; VCAM1, Vascular cell adhesion protein-1; VEGF, Vascular endothelial growth factor.

Many aspects of PRES in pediatric oncologic/hematologic diseases—i.e., clinical features, prognostic factors and outcome, and management—remain unclear. Diagnostic criteria for PRES have been proposed by previous studies (17, 19, 33, 47). PRES is a neurotoxic state that manifests during oncologic/hematologic treatment (10, 11, 15, 18, 24, 25, 27, 32, 48–54); higher single and cumulative doses of chemotherapy and HSCT and longer treatment duration are associated with greater neurotoxicity to both central and peripheral nervous systems (CNS and PNS, respectively) (52, 54). Cytotoxic therapy may contribute to PRES by directly acting on the vascular endothelium and causing capillary leakage and destruction of the blood–brain barrier (33, 55). In ALL patients, PRES mainly occurred during the induction phase of chemotherapy with methotrexate, prednisolone, vincristine, doxorubicin, and asparaginase (11, 12, 24, 26, 36, 46, 56, 57). Vincristine was been linked to peripheral neuropathy and may be a causative factor in PRES (43, 51–54, 58). Additionally, asparaginase, methotrexate, cytarabine, and intrathecal chemotherapy are known to be neurotoxic to the CNS and PNS (52, 59). High-dose chemotherapy and drugs used to prevent GVHD in HSCT (59) including cyclosporine, and tacrolimus can lead to PRES (52, 59) possibly by promoting hypertension (60), similar to the cytotoxic steroid-based drugs that are typically included in chemotherapy/HSCT regimens (61–64). Recent studies indicate that steroids promote PRES in patients with oncologic/hematologic diseases (11, 15, 52, 64, 65) (Figure 4).

Electrolyte disorders are common in cancer patients—occurring in as many as one-third—and may worsen prognosis (82–86). The manifestations of acute hyponatremia vary from non-specific symptoms (e.g., headache, nausea, vomiting, and muscle cramps) to life-threatening conditions such as bradycardia, hypertension, impaired thermoregulation, cerebral herniation, convulsions, and coma (82, 83, 87). HSCT and chemotherapy-induced febrile neutropenia—which is associated with decline in blood electrolyte (sodium, potassium and magnesium) levels—have a potentially fatal outcome. Thus, it is critical to monitor electrolyte balance in cancer patients (88, 89). On the other hand, electrolyte abnormalities are useful prognostic indicators in palliative care (90). PRES-related electrolyte disorders are rare, although there is increasing evidence that hyponatremia contributes to the pathogenesis of PRES (27, 42, 91, 92); the mechanism may involve interference by aquaporins with the regulation of osmotic pressure in the brain (93–95). Hyponatremia was observed in 70.5% of ALL patients with PRES treated with chemotherapy along with hypocalcemia (41.9%) and abnormal magnesium (25.6%) and glucose (35.7%) (11), as well as in 38% of patients who underwent HSCT (96). A case of ALL with PRES secondary to hyponatremia has also been reported (42). In our pediatric cohort with oncologic/hematologic diseases, elevated blood sodium, potassium, and magnesium levels were protective factors against PRES, implying that interventions that increase blood electrolyte concentrations can be beneficial in this group (Figure 4).

Although the exact cause of PRES is not known, it is thought to be related to the production of toxins induced by HSCT and chemotherapy that target capillary endothelial cells, leading to the failure of cerebral blood pressure autoregulation, endothelial dysfunction, and vasogenic edema (52, 97). PRES is usually observed in the context of acute hypertension (sometimes treatment-induced) (10–12, 15, 17–19, 22–28, 32, 33, 44, 46, 47, 57, 65, 98–105), and may be overlooked in patients with near-normal blood pressure at symptom onset (98–100). Hypertension is more common in children than in adults with PRES (106). Between 67 and 100% of patients with PRES have hypertension after undergoing HSCT or receiving chemotherapy in pediatric oncologic/hematological diseases (10–13, 15, 24–28, 36, 40, 46, 56, 57, 105, 107, 108). In our systematic review, the rate of hypertension was higher after HSCT than after chemotherapy (75.0 vs. 51.0%) in pediatric patients with oncologic/hematologic diseases and PRES; we previously showed that hypertension leads to a poor outcome in this group (10) (Figure 4).

Adverse events associated with blood transfusion in cancer patients following chemotherapy/HSCT include febrile non-hemolytic transfusion, allergic, and delayed hemolytic transfusion reactions; acute hemolytic transfusion reactions (AHTRs); transfusion-associated circulatory overload or acute lung injury (TACO and TRALI, respectively), GVHD; dyspnea; immunomodulation; red blood cell alloimmunization; iron overload; and microbial infection. TRALI, TACO, and AHTRs are potentially fatal complications (68). TACO is characterized by respiratory distress, pulmonary edema, left or right heart failure, elevated central venous pressure, or hypertension, which occur within 2 h or up to 6 h after the start of transfusion (109). Elevated blood pressure is a risk factor for TACO (69). The rapid increases in hemoglobin level and blood viscosity after transfusion are thought to cause PRES by inducing acute vascular endothelial dysfunction and increasing vascular resistance, resulting in extravascular leakage of fluid and macromolecules in the brain (70). There have been several reports of blood transfusion-related PRES, with symptoms lasting from 2 h to over 1 month (70, 75). Only a few studies have investigated risk factors for PRES related to transfusion in children; these involved patients with sickle cell disease (SCD) (110) or thalassemia (15). Ours is the first report of blood transfusion-related PRES in pediatric oncologic/hematologic diseases (Figure 4).

There is no specific intervention for PRES, but the condition is reversible by addressing the etiology. Clinical management involves a combination of symptomatic life-supporting treatments and control of causative factors. A previous retrospective study found that mechanical ventilation was required in 71% of adult patients with severe PRES; the mortality rate attributed to PRES was 5.7%, with toxicity (44%) and hypertensive encephalopathy (41%) being the main causes of death (47). In pediatric patients with oncologic/hematologic diseases, early diagnosis of PRES is critical for avoiding neurologic sequelae and death (10–12, 15, 17, 19, 24, 32, 105, 111). PRES in children is more common in hematologic diseases compared to other malignancies and is associated with hypertension, infection, and steroid use; seizures are the most common acute manifestation. Most MRI changes resolve, but persistent imaging abnormalities and epilepsy can develop (44). We previously demonstrated that female sex, age >10 years old, acute GVHD, hypertension, immunodeficiency, SCD, T cell leukemia, and CNS leukemia/involvement are linked to poor outcome in children with oncologic/hematologic diseases and PRES (10); in this population, the main causes of death are underlying diseases, severe infection, MODS, respiratory failure, GVHD, and severe organ toxicity (15, 18, 25–32), although in some cases mortality was a direct result of PRES (12, 18, 24, 28, 30). In our systematic review, only 6.8% of the deaths were attributable to PRES, and the mortality rate was higher following HSCT than chemotherapy (25.3 vs. 7.7%), indicating that the latter is a safer treatment option for pediatric patients with oncologic/hematologic diseases who develop PRES. Based on the above findings, we recommend the following protocol for the management of PRES in pediatric patients with oncologic/hematologic diseases treated with chemotherapy or HSCT: treatment of specific symptoms including seizures, lowering of blood pressure, and eliminating or reducing causative factors/medications (111–114) (Table 5).

**Table 5:** Management or treatment protocol for PRES in pediatric oncologic/hematologic diseases.

**Consensus/factors**	**Management/treatment (recommendations)**	**References**
General symptomatic treatment	1. Monitoring of airways and ventilation; intubation (if insufficient oxygenation) 2. Maintenance of hydration (intravenous crystalloid fluids), adequate arterial oxygenation, correction of electrolyte disturbances (blood sodium, potassium, and magnesium), routine blood examination (platelets and hemoglobin), other complications (fever, acid-base balance, cognitive impairment, infection, etc.) 3. Insertion of central venous catheter (if cardiac dysfunction); consider transferring to pediatric intensive care unit if patient is critically ill	(12, 111)
Elimination or notable diminution of causative factors/medications (toxicity management)	1. Recognition of neurotoxicity is important to prevent further neurologic injury and to distinguish this toxicity from nervous system involvement in cancer 2. “Stop-and-go” regimens (chemotherapies) may be associated with lower neurotoxicity 3. Longer infusion (hydration and alkalization) duration may reduce neurotoxicity 4. Manage treatments of longer duration, which have increased risk of neurotoxicity 5. Decrease or stop single doses of chemotherapy/HSCT/immunosuppressants (methotrexate, vincristine, asparaginase, cytarabine, steroid, cyclosporine, tacrolimus, ifosfamide, cyclophosphamide, rituximab, etc.) or regimens [induction, high-dosage regimens, intrathecal, HSCT (chemotherapy), etc.] 6. Other risk factors (GVHD, transfusion management, etc.)	(12, 31, 48, 50–54, 59, 111, 115, 116)
Lowering of blood pressure	1. Blood pressure goal: <13 or ≥13 years, 130/80 mmHg, <90th percentile or <130/80 mmHg, whichever is lower; recommendations for 24-h ambulatory blood pressure monitoring 2. Intravenous therapy preferred: (1) diuretics (furosemide at 1–2 mg/kg, mannitol at 0.5–2 g/kg); (2) nicardipine at 1–3 μg/kg; esmolol at 0.05–0.3 mg/kg; sodium nitro prussiate at 0.5–8 μg/kg 3. Oral treatment: nifedipine at 0.25 mg/kg, isradipine at 0.05–0.1 mg/kg, captopril at 0.1–0.2 mg/kg	(12, 111–113)
Treatment of status epilepticus (intravenous anticonvulsants)	1. First line: lorazepam at 0.1–0.3 mg/kg (maximum 4 mg) IV, may repeat dose; midazolam at 0.1–0.2 mg/kg (maximum 10 mg) IM single dose 2. Second line: levetiracetam at 10–60 mg/kg (maximum 4,500 mg) IV single dose; valproic acid at 10–40 mg/kg (maximum 3,000 mg), IV single dose 3. Third line: phenobarbital at 10–20 mg/kg (maximum 1,000 mg) IV; midazolam at 0.1–0.2 mg/kg (maximum 10 mg), infusion 0.1–0.2 mg/kg/h	(12, 111, 114)

*GVHD, graft-vs.-host disease; HSCT, hematopoietic stem cell transplantation; IV, intravenous injection; IM, intramuscular injection*.

Our systematic review of 21 studies including 1,213 participants and our 20 cases provides the most comprehensive analysis to date of PRES in children with oncologic/hematologic diseases. However, there were several limitations to the current work. (1) Selection bias could not be ruled out in our comparative analysis of factors related to PRES in oncologic/hematologic and non-oncologic/hematologic diseases, as we included adults in the latter group because of the scarcity of pediatric patients. (2) Given the observational study design, we could not exclude the possibility of confounding factors, although there was consistency between the primary and propensity factor-matched analyses. Nevertheless, we were unable to establish a cause-and-effect relationship between PRES and oncologic/hematologic diseases as some of our patients were lost follow-up and there was no radiologic follow-up. (3) There may have been publication bias in our meta-analysis because of restrictions on the year of publication. (4) Some of the included case series had insufficient patient information, corresponding to a low level of evidence. (5) In our previous study, we identified several factors associated with a poor outcome for PRES in pediatric oncologic/hematologic diseases (10); however, the random-effects model in the present study identified only 2 of these factors (hypertension and blood transfusion) as being significantly associated with PRES, indicating low concordance between the findings of the 2 studies.

## Conclusions

The results of our study identified hypertension; blood transfusion; and severe decreases in blood sodium, potassium, and magnesium as risk factors for PRES in pediatric patients with oncologic/hematologic diseases. Neurotoxicity related to chemotherapy and HSCT was related to a longer treatment duration. PRES was more common with HSCT compared to chemotherapy and had a nearly 2 times higher mortality rate in patients with oncologic/hematologic diseases than in those with other types of disease. Knowing the risk factors and protective factors based on the characteristics of the individual patient can help to prevent neurological complications or improve their management.

## Data Availability Statement

The original contributions presented in the study are included in the article/Supplementary Material, further inquiries can be directed to the corresponding author.

## Ethics Statement

The studies involving human participants were reviewed and approved by the Second Xiangya Hospital, Hunan Children's Hospital, Hunan Provincial People's Hospital. Written informed consent to participate in this study was provided by the participants' legal guardian/next of kin. Written informed consent was obtained from the individual(s), and minor(s)' legal guardian/next of kin, for the publication of any potentially identifiable images or data included in this article.

## Author Contributions

MH, MX, JT, and CW: study conception and design and manuscript writing. MH, PH, MX, HL, and RP: development of methodology and statistical analysis. JT, ZS, FW, CL, AA, HL, PW, KC, and WW: data collection. All authors manuscript formatting and editing.

## Conflict of Interest

The authors declare that the research was conducted in the absence of any commercial or financial relationships that could be construed as a potential conflict of interest.
