# Supplementary material for: Management and Clinical Outcome of Posterior Reversible Encephalopathy Syndrome in Pediatric Oncologic/Hematologic Diseases: A PRES Subgroup Analysis With a Large Sample Size
Source: Front Pediatr. 2021 Jul 1;9:678890. doi: 10.3389/fped.2021.678890 (PMC8280768; doi:10.3389/fped.2021.678890)
Supplement: Supplementary file 1 [file Data_Sheet_1.pdf]

Refer(s)	Age(Ys)/ Sex	Primary diagnosis	Oncological treatment at PRES onset	PRES related				Neuroimaging related			patient prognosis
				EEG findings/ Time of treatment to PRES	Symptoms/Sign	Treatment		Initial lesion site	Follow up findings		
						Anti-epileptic	Anti-HT		Time Of Follow up	Outco me	
Our case	2.1/F	B-ALL	CTX, Arc-C, IT	NA	Sei, Vis, Vom, Men, Hyp	Dia, PB	Nif, Diu, SNP	CT: (Wm); MRI: (Pa)	NA	Normal	Alive
Our case	10/M	NHL	Dex, VCR, Ifo, DNR, HD-MTX, L-Asp, IT	DS, Pa, Oc	Sei, Vom, Men, Hyp	Mid	Am, Dui	MRI: (Pa, Oc)	NA	NA	Dead
Our case	6.2/F	B-ALL	VDLD	FS	Hea, Vis, Vom	NA	Dui	MRI: (Fr, Te, Pa, Wm)	NA	NA	Alive
Our case	3.2/M	B-ALL	VDLD	FS	Sei, Vom, Hyp	Dia	Dui	MRI: (Oc)	D+30	CR	Alive
Our case	6.9/F	HPS	VP16, Dex, CsA	FS	Sei, Vom, Men, Hyp	Dia	Nif, Am, Dui	MRI: (Cb, Oc)	D+23	PR	Lost follow up
Our case	16/F	AML	HSCT, CsA	NA	Men, Sei, Hyp	Dia, Mid, VPA, PHT	Nit, Dui	MRI: (Fr, Pa, Te, Oc); CT: (Bg, Oc)	D+26	RL	Lost follow up
Our case	8.8/M	NHL	VCR, CTX, Pre	NA	Hea, Men, Sei, Vis, Hyp	Dia, Mid	Nif, SNP, Dui	MRI: (Th, Hi, PV); CT: (Pa)	D+40	PR	Alive
Our case	4.9/M	B-ALL	6MP, MTX, Dex, VDS, IT	DS, Oc	Sei, Men	PB	Oxi, Nit, Diu	MRI: (Fr, Wm)	M+4	RL (Fr)	Alive
Our case	6.8/M	NHL	Dex, VCR, MTX, CTX, DNR, IT	FS	Vom, Hyp	NA	Cap, Am	MRI: (Pa, Wm, Oc); CT: (Pa, PV)	D+33	PR	Alive
Our case	6.8/M	NHL	MTX, CTX, DNR, VCR, Pre, IT	NA	Sei, Men, Vis, Hyp	Dia, Mid	Diu	MRI: (Pa,Te); CT: (Pa)	D+60	RL	Alive
Our case	6.6/M	B-ALL	6MP, MTX, Dex, VCR, IT	Normal	Sei, Vis, Men	Dia, Mid	Dui	MRI: (Wm); CT: (Wm)	NA	NA	Alive
Our case	6/M	B-ALL	CTX, Arc-C, 6MP, IT	NA	Vom, Sei, Men, Hyp	Dia, PB, Mid	Oxi, Dui	MRI: (PV, Pa, Oc)	NA	NA	Alive
Our case	4.4/M	B-ALL	CTX, Arc-C, 6MP, IT	Normal	Sei, Men	Dia, PB, Mid	Oxi, Dui	MRI: (Pa, PV)	D+30	PR	Alive
Our case	7.1/F	B-ALL	inductionVDLD	FS	Hea, Sei, Men	Dia, Mid	Oxi, Dui	CT: (Ch); MRI: (Pa)	D+120	CR	Alive
Our case	11/M	B-ALL	CTX, Arc-C, 6MP, IT	DS	Sei, Hyp	Dia	Nif, Dui, Irb	CT: (Oc, Fr, Pa)	D+6	PR	Alive

Our case	9.1/M	B-ALL	inductionVDLD	NA	Sei, Vis, Hyp	Dia	Nif, Dui	CT (-); MRI: (Te, Oc)	D+58	CR	Alive
Our case	6/M	B-ALL	inductionVDLD	NA	Sei, Vis, Hyp	Dia	Nif, Dui	MRI: (Fr, Pa, Oc)	NA	NA	Alive
Our case	9.1/M	B-ALL	HR-1	FS	Sei, Men, Vis, Vom, Hyp	Dia, PB, Mid	Oxi, Dui	CT: (Pa, Oc)	D+3	Same	Alive
Our case	13.3/F	HPS	CsA, MP, VP-16	FS	Sei, Vis, Men, Hyp	Dia, PB	Nif, Dui, Am	CT(-); MRI: (Fr, Pa, Oc, Wm)	D+5;D+24	PR; CR	Alive
Our case	3.1/M	NB	THP, CTX, VCR	NA	Sei, Men, Vom	PB, Mid	Dui	MRI: (Pa, Oc)	NA	NA	Death
Eroglu(1) 2017 Turkey	14/M	B-ALL	VDLD	NA	Sei, Vis, Men	Mid, PHT	NA	CT: (-); MRI: (Pa, Wm, Fr, Oc, CB)	D+15	CR	Alive
Cifci(2) 2019 Turkey	9./F	B-ALL	VDLD	NA	Vis, Men	Yes(NA)	Diu	MRI: (-)	NA	Normal	Alive
	12/M	T-ALL	VDLD	NA	Sei, Men, Hyp	Yes(NA)	Yes(NA)	MRI:(Pa, Oc, Fr)	NA	CR	Alive
	8/F	T-ALL	ReinductionVDLD	NA	Vom, Sei, Hyp	NA	NA	NA	NA	CR	Alive
	12/M	B-ALL	VDLD	NA	Sei, Vis	Yes(NA)	Yes(NA)	MRI: (Pa, Oc, Fr)	NA	CR	Alive
	5/M	B-ALL	VDLD	NA	Men, Sei, Hyp	Yes(NA)	Yes(NA)	MRI: (Pa, Oc, Fr)	NA	RL	Alive
	6/F	B-ALL	VDLD	NA	Men, Hyp	NA	NA	MRI: (Wm)	NA	CR	Alive
	6/F	B-ALL	VDLD	NA	Sei	NA	NA	MRI: (Fr)	NA	CR	Alive
	0.5/F	B-ALL	VDLD	NA	Sei, Hyp	Yes(NA)	Yes(NA)	MRI: (PV)	NA	RL	Alive
	8/M	B-ALL	ReinductionVDLD	NA	Sei	Yes(NA)	NA	MRI: (Pa, Oc)	NA	CR	Alive
	10/M	B-ALL	Consolidation	NA	Vis, Sei, Men	Yes(NA)	NA	MRI: (-)	NA	Normal	Alive
	6/M	B-ALL	VDLD	NA	Vom, Sei, Men	Yes(NA)	Yes(NA)	MRI: (Wm, BG)	NA	CR	Alive
	4/F	B-ALL	VDLD	NA	Sei, Men, Hyp	Yes(NA)	Yes(NA)	NA	NA	CR	Alive
	14/M	T-ALL	Consolidation	NA	Sei, Men, Hyp	Yes(NA)	Yes(NA)	MRI: (consistent with PRES)	NA	CR	Alive
	8/F	B-ALL	VDLD	NA	Vis, Sei, Hyp	Yes(NA)	Yes(NA)	n/a	NA	CR	Alive
Kapoor(3) 2018 India	7/M	Tha	HSCT, CTX, Bu, MTX	D+15	Hea, Sei, Men	NA	Nif	CT: (Oc)	D+100	CR	Alive
	6/F	Tha	HSCT, CTX, Bu, MTX	D+3	Vis, Sei, Hyp	NA	Lab	MRI: (Oc, Pa, Fr)	D+270	CR	Alive
	9/M	AA	HSCT, Flu, CTX, Tac, Mmf, storoids	D+27	Hea, Sei, Men, Hyp	NA	Lab	MRI: (Oc, Pa)	D+74	CR	Dead

	11/M	Tha	HSCT, CTX, Bu, MTX	D+34	Vis, Sei, Hyp	NA	Lab	MRI: (Oc)	D+60	RL(Hemianopia)	Alive
	11/M	Tha	HSCT, Tre, CTX, storoids	D+11	Hea, Men, Hyp	NA	Lab	MRI: (Fr, Te, Oc)	D+150	CR	Alive
	7/M	CDA	HSCT, CTX, Bu, MTX	D+60	Vis, Men, Hyp	NA	Lab	MRI: (Fr)	D+90	RL (Aphasia)	Alive
Khan(4) 2017 Pakistan	M	ALL	Dex, VCR, P-Asp	NA	NA	Lev	Yes (NA)	MRI: (PC)	NA	NA	Alive
	M	ALL	Dex, VCR, P-Asp	NA	NA	Lev	NA	MRI: (PC, SR)	NA	NA	Alive
	M	BL	CTX, Dox, Pre, VCR	NA	NA	NA	Yes (NA)	MRI: (PC, AC, SR)	NA	NA	Dead
	F	ALL	Dex, VCR, P-Asp	NA	NA	Lev	Yes (NA)	MRI: (PC, AC, SR)	NA	NA	Alive
	F	BL	CTX, Pre, VCR	NA	NA	Lev	NA	MRI: (PC, AC, SR)	NA	NA	Dead
	M	ALL	6MP, MTX	NA	NA	Lev	Yes (NA)	MRI: (PC)	NA	NA	Dead
	M	DLBCL	steroids	NA	NA	PHT	NA	MRI: (PC)	NA	NA	Alive
	M	BL	CTX, Pre, VCR	NA	NA	PHT	NA	MRI: (PC)	NA	NA	Dead
	M	HL	Pre	NA	NA	Lev	NA	MRI: (AC, PC)	NA	NA	Alive
	M	DLBCL	CTX, Dox, Pre, VCR	NA	NA	NA	NA	MRI: (PC, AC, SR)	NA	NA	Alive
	M	T-LBL	Dex,L-Asp, DNR, VCR	NA	NA	Lev, PHT	NA	MRI: (PC, AC, SR)	NA	NA	Alive
	M	DLBCL	Ifo, Car, VP16	NA	NA	Lev	Yes (NA)	MRI: (PC)	NA	NA	Dead
	M	ES	Ifo, VCR, Dox, VP16	NA	NA	No	NA	MRI: (PC, SR)	NA	NA	Dead
	M	BL	CTX ,Pre, VCR	NA	NA	PHT	Yes (NA)	MRI: (AC, PC)	NA	NA	Dead
	M	HL	Pre	NA	NA	PHT	NA	MRI: (AC, PC)	NA	NA	Alive
	M	Re-ALL	CTX, Arc-C	NA	NA	Lev	Yes (NA)	MRI: (AC, PC)	NA	NA	Dead
	F	Re-ALL	VCR, P-Asp	NA	NA	Lev, PHT	NA	MRI: (PC)	NA	NA	Alive
	M	ALL	Dex, VCR, P-Asp	NA	NA	Lev	Yes (NA)	MRI: (PC)	NA	NA	Alive

	F	BL	CTX, Dox, Pre, VCR	NA	NA	Lev	Yes (NA)	MRI: (PC)	NA	NA	Dead
Fraint(5) 2017 USA	3/F	B-ALL	VDLD	NA	Hyp, Vis	NA	Ena	MRI: (Oc, Pa)	M+90	CR	Alive
Tavil(6) 2016 Turkey	10/M	AML	HSCT, Sor, Clo, Arc-C, IT	D+14	Hea, Sei	Mid, PHT, Lev	NA	MRI: (Wm, Pa, Oc)	NA	NA	Dead
Veljanovska (7) 2019 Macedonia	7/F	MDS	HSCT, CTX, Bu, CsA, MP, Tac	D+34	Sei, Hea, Vis, Men, Hyp	Yes (NA)	Yes (NA)	MRI: (high suspicion for PRES)	D+53	RL	Dead
Pavlidou (8) 2016 Greece	13/M	BL/L	Dex, Ifo, CTX, MTX, VCR, VM26, Dox, IT	D+90	Sei, Hea, Hyp	Dia, PHT	Dui	MRI: (Fr, Pa, Oc)	D+14	CR	Dead
Aureli(9) 2017 Germany	2/M	NB	VCR, DNR, CTX	NA	Sei, Hea, Men, Hyp	Yes (NA)	Yes (NA)	MRI: (Oc, Pa)	D+14	CR	Alive
Musiol(10) 2017 Poland	8.5/F	AML	Arc-C	EEG(+, NA), D+150	Sei, Hea, Men, Vis, Hyp	Yes (NA)	Yes (NA)	MRI: (+, NA)	Y+5	CR	NA
	13/F	HB	Cisplatin	EEG(+, NA), D+66	Sei, Hea, Men, Vom, Hyp	Yes (NA)	Yes (NA)	MRI: (+, NA); CT:(+, NA)	Y+5	CR	Dead
	3.3/M	WT/ Nephr	HSCT, VCR, Car, storoids	EEG(+, NA), D+556	Sei, Hea, Men, Vis, Hyp	Yes (NA)	Yes (NA)	CT: (+, NA)	Y+5	CR	NA
	11/M	ALL	VCR, Arc-C, MTX P-Asp, storoids	EEG(+, NA), D+55	Sei, Men, Hea	Yes (NA)	Yes (NA)	MRI: (+, NA); CT:(+, NA)	Y+5	CR	NA
	3.3/M	NB	Cisplatin	D+84	Hyp, Hea, Vis	Yes (NA)	Yes (NA)	MRI: (+,NA)	Y+5	CR	NA
	8.5/M	PNET	Steroids	D+0	Hyp, Hea, Sei, Men, Vom	NA	Yes (NA)	MRI: (+, NA)	Y+5	CR	NA

	2.8/M	ALL	VCR, DNR, steroids, P-Asp, MTX	D+67	Hyp, Vom	Yes (NA)	Yes (NA)	MRI: (+, NA)	Y+5	CR	NA
	16/F	AA	CsA, steroids	D+6	Sei, Hea, Men, Vis, Hyp	Yes (NA)	Yes (NA)	MRI: (+, NA)	Y+5	CR	NA
Shkalim-Zemer(11) 2017 Israel	8.4/M	Tha	HSCT, CTX, Bu, CsA	D+18	Men, Hea, Vom, Vis, Sei, Hyp	NA	NA	MRI: (Oc, SC)	M+9	CR	Dead
	2.2/M	CPRCA	HSCT, Flu, Tre, TT, ATG, CsA, MTX	D+32	Hea, Sei, Hyp	NA	NA	MRI: (PV)	NA	NA	Dead
	4.5/M	CT	HSCT, Flu, Mel, TT, ATG, CsA	D+32	Men	NA	NA	MRI: (PV, SC)	M+17	CR	Alive
	8.5/M	AA	HSCT, ATG, CTX, MTX	D-1	Sei, Hea, Hyp	NA	NA	MRI: (Oc)	M+3.5	CR	Alive
	14/F	CML (BC)	HSCT, VP16, TBI, CsA	D+15	Vis, Sei, Hyp	NA	NA	MRI: (PV)	M+3.5	PL(Wm, Pa)	Alive
	8.2/M	Tha	HSCT, Flu, TT, Tre, CsA, MTX	D+53	Sei, Hyp	NA	NA	MRI: (Pa, Oc)	NA	NA	Alive
	7.8/F	Re-ALL	HSCT, VP16, TBI, ATG, CsA, MTX	D+15	Vis, Hea, Sei, Vom, Hyp	NA	NA	MRI: (Pa, Oc, Fr, PV)	NA	NA	Dead
	5.5/M	ALL	HSCT, VP16, TBI, ATG, CsA, Pre	D+23	Men, Hyp	NA	NA	MRI: (SC, Oc, Fr)	NA	Lost follow up	NA
	12/F	AMBL	HSCT, Flu, Mel, CsA, MTX	D+37	Sei, Hyp	NA	NA	MRI: (Te, Pa)	NA	NA	Dead
	10/M	CDA	HSCT, Flu, TT, Tre, CsA, MMF	D+70	Men, Hea, Sei, Hyp	NA	NA	MRI: (Oc, Fr)	M+4	PR	Dead
	5.7/F	AML	HSCT, ATG, Flu, Tre, CsA	D+81	Sei, Hyp	NA	NA	MRI: (Oc, Fr, SC)	M+3	CR	Dead
	17/M	T-ALL	HSCT, Flu, TT, Tre, Tac	D+158	Men, Hyp	NA	NA	MRI: (Oc)	NA	NA	Dead
	4/M	AML	HSCT, ATG, Bu, CTX, Pre	D+210	Sei	NA	NA	MRI: (Oc)	NA	NA	Dead

	9.7/M	AML	HSCT, TBI, Flu, CTX, CsA, Pre	D+88	Hea, Sei, Hyp	NA	NA	MRI:(Oc, Wm, Fr, Pa)	NA	NA	Alive
Danhofer (12) 2019 Czech Republic<br><br>F 11/21	17	T-ALL	MTX, CTX, 6MP, VCR, Arc-C, DNR, L-Asp, steroids	normal	NA	NA	NA	MRI: (Pa, Oc)	D+12	CR	Dead 3 (14.3%)
	8	B-ALL	MTX, VCR, DNR, steroids, L-Asp	FS, Pa, Oc	NA	Dia	Dui	MRI: (Pa, Oc)	D+12	CR	
	6	ALL	MTX, VCR, DNR, CTX	FS, Oc	NA	Dia	Ena	CT: (Pa, Oc)	D+60	CR	
	7	B-ALL	MTX, VCR, DNR, steroids, L-Asp	normal	NA	No	Cap, Isr	MRI: (Fr, Oc)	D+60	CR	
	4	ALL	MTX, CTX, 6MP ,VCR, Arc-C, DNR, L-Asp, steroids	FS, Oc	NA	Dia	NA	CT: (Pa, Oc)	D+14	CR	
	2	ALL	MTX, CTX, 6MP, Arc-C, L-Asp, steroids	FS, Te, Pa, Oc	NA	Dia	NA	MRI: (Pa, Oc)	D+60	CR	
	4	ALL	MTX, Arc-C, CTX, 6MP	PLEPD, Pa,Te	NA	Dia, Lev	Ena	MRI:(Fr, Te, Pa, Oc, CB)	D+60	CR	
	3	B-ALL	Steroids	normal	NA	No	Dui	MRI: (Pa, Oc)	D+14	CR	
	6	T-ALL	MTX, VCR, DNR, L-Asp, steroids	FS. Oc	NA	Dia, Lev	Ena, Dui, Pro	MRI: (Oc)	D+14	CR	
	16	ALL	MTX, VCR, CTX, 6MP, DNR, L-Asp, steroids, Arc-C	FS. Oc	NA	Dia, VPA	Cap, Dui	MRI: (Oc)	D+14	CR	

	5	AML	MTX, VCR, Pre, VP16	DS. Oc	NA	NA	Dih, Dui, Clo, Ena, Aml	MRI: (Fr, Te, Pa, Oc)	D+90	CR	
	7	B-ALL	MTX, VCR, DNR, L-Asp, steroids	PLEPD, Pa, Oc	NA	Dia, Lev	Cap, Isr	MRI: (Pa, Oc)	D+14	CR	
	4	ALL	MTX, VCR, DNR, L-Asp, steroids	DS, Te, Pa, Oc	NA	Dia, PB, Lev	Dih, Dui, Ena	MRI: (Pa, Oc)	D+21	CR	
	3	ALL	MTX, VCR, 6MP, L-Asp	normal	NA	Dia, Lev	Ena	MRI: (Fr, Pa)	D+60	CR	
	7	NHL	MTX, VCR, L-Asp	FS, Oc, Te	NA	Lev, VPA	Dih, Clo, Ena, Met	MRI: (Pa, Oc)	D+28	CR	
	5	AA	ATG, CsA, steroids	FS, Fr,Te, Pa, Oc	NA	Lev	Dih, Dui, Clo, Ena, Am	MRI: (Pa, Oc)	D+14	CR	
	8	BL	MTX, VCR, CTX, DNR, Arc-C, Rit, steroids	FS. Pa	NA	Lev	Ena	MRI: (Pa)	D+90	CR	
	13	B-ALL	MTX, CTX, 6MP, VCR, Arc-C, DNR,steroids	FS, Pa, Oc	NA	CLZ, Lev	Clo, Met, Ena	MRI: (Fr, Te, Pa, Oc)	D+28	CR	
	10	CML	HSCT, ATG, CTX, Flu	DS	NA	CLZ, Lev	Dih, Ler, Per, Met, Cap	MRI: (Fr, Pa, Oc)	D+60	CR	
	7	ALL	MTX, VCR, DNR, L-Asp, steroids	PLEPD, Te, Oc	NA	CLZ, Lev	Dih, Ena, Dui	MRI: (Oc)	D+90	RL (Oc)	
	3	B-ALL	MTX, VCR, DNR, L-Asp, steroids	FS, Te, Oc	NA	Dia, Lev	Dih, Clo, Ena, Pro	MRI: (Fr, Te, Pa, Oc, CB)	D+90	RL (Pa, Oc)	
Tambosco(13) 2016 Italy	8/F	Re-AML	HSCT, CsA, MP	D+36	Men, Hyp, Sei	Dia	CCB	MRI: (Pa, Oc, SC)	M+4	PR	Dead
	4/F	AA	HSCT, CsA, MTX	D+45	Vis, Hyp	NA	Lab	MRI: (Te, Oc, BG);CT: (BG)	Y+2	CR	Alive

	12/F	ALL	HSCT, CsA, MP, ATG	D+45	Sei, Vis, Hyp	Dia, VPA	ACE-I, Ram	CT: (+); MRI: (CB, Te, Pa)	M+1	CR	Dead
	7/M	Re-AML	HSCT, CsA	D+1	Hea, Vom, Men, Sei, Hyp	Lor	Nif, CCB, ACE-I	CT: (+); MRI: (CB, Te, Fr, Oc, Pa)	M+2	CR	Alive
	9/M	Re-ALL	HSCT, CsA, MTX	D+45	Men, Vis, Hyp	NA	CCB, Am	MRI: (Fr, Oc)	D+7	RL(Fr, Oc)	Dead
Hafez(14) 2020 Egypt	> 10y (31/50); F, 27/50	ALL 19; AML 5; Lym 8; SL 13; postBMT 5	Chemo 40; VDLD 12; Maintaince 4; LMB protocol 7; High-dose block 6; Calcineurin inhibitor and 4	NA	Sei 45 (90.0%); Men 34 (68.0%); Vis 8 (16.0%); Hea 14 (28.0%); Hyp 43 (86%)	NA	NA	Oc 46/50; Pa: 37/50; Te: 11/50; Fr: 16/50; Bg: 4/50; CB: 6/50; Th: 3/50	2-4W; >3M	CR: 30 (60%); PR: 8 (16%); PL: 5 (10%)	Dead: 5/50 (10%); (2 PRES related; 3 related to other causes)
Lin(15) 2019 China	Median 7.0y; F 7/13	B-ALL 13	VDLD 13	17<D<34	Sei 13 (100%); Vis 4 (31.0%); Men 2 (15.0%); Hyp (NA)	NA	NA	Oc 12 (92.3%); Fr 7 (53.8%); Te 5 (38.4%); Pa 3 (23.1%)	M+1 3(23.1%), CR ; >1M 10 (78%) PR		Dead 1/13 (1.1%), related to CNS infection
Li(16) 2018 China	Median 4.0y; F 6/11	Tha 11	HSCT 11; steroid 10/11	NA	Hyp: 10 (91.7%) ; Sei: 7 (63.6%); Men: 11(100%); Hea: 7 (63.6%); Vom: 6 (54.54%); Vis: 1 (1.0%)	NA	NA	NA	NA	NA	Dead 2/11 (18.2%). Related to servere infection
Anastasopoulou(17) 2018 Sweden	>10y: 16/52 F: 27/52	ALL: 52; B-ALL: 37; T-ALL: 15	Induction 28/52; consolidation 10/52; high-risk block 6/52	EEG 31/52; FS 16; DS 13	Hyp 41/52 (78.8%) ; Sei 43/52 (82.7%); Men 33/51(64.7%); Hea 15/51 (29.9%); Vom 10/50 (20.0%); Vis 17/51 (33.3%)	Antiepileptics 36/48; antihypertensives 33/49; treatment at ICU 33/51; Changes in chemotherapy 32/51		Oc 29/48; Pa 27/48; Fr 19/48; Te 12/48; Cb 5/48; Bg 4/48	Median time from the first MRI 33days; CR 17/36		Dead 2/52 (3.8%); related to servere organ toxicities and infection
Khan(18) 2016 USA	F 16/37	Leuk 25; SL 12; Re-leuk 8	Chemo 21; BMT 12; Steroid 29; Other 4	EEG 23/37; FS 14/23 DS 6/23	Hyp 35 (95%) ; Sei 15/37; Men 25/37; Hea 15/37; Vis 10/35	Benzodiazepines 26/37; phenytoin 14/37; levetiracetam 22/37; Antiepileptics 12/37		Oc 36/36; Pa 27/36; Fr 20/36; Te 17/36; Cb 8/36; Bg 2/36	NA	NA	NA
Gaziev(19) 2017	>10y 14/31; F 8/31	Tha 18; SCD 13	HSCT 31	FS 23/31;	Hyp 31/31; Sei 30/31; Men 20/31;	NA	NA	Oc 13 (42%); Pa 10 (32%); Fr 14 (45%); Te 15 (48%); Cb 1 (3)	NA	NA	Dead 7/31 (22.6%); related to GVHD

Italy				DS (θ) 15/31; DS (δ) 17/31	Hea 28/31; Vis 12/31; Vom 18/31						
Chen(20) 2020 China	Median 11y; F 9/22	AA 3/22; AML 6/22; ALL 6/22; CML 3/22; MDS 3/22; Lym 1/22	HSCT 22	NA	Hyp (NA); Sei 21/22; Men 1/22; Hea 2/22; Vis 4/22	NA	NA	Oc 16/22; Pa 15/22; Fr 5/22; Te 5/22; Cb 2/22; Th 3/22	NA	NA	Dead 3/22 (13.6%); 1 severe irreversible PRES; 2 infection and GVHD
Zama(21) 2018 Italy	Median 9.2y F 53/112	Leuk 81 (ALL 60+AML 14); Lym 7(NHL 7); ST 11; Non-MD 20	Chemo 67/112; HSCT 42/112; Other 3/112	EEG(+, 95/122);(-, 6/112); (NA, 11/112)	Hyp 84/112; Sei 97/112; Men 45/112; Hea 15/112; Vis 25/112	Benzodiazepines 81/112; phenytoin 10/112; Phenobarbital 20/112		NA	NA	NA	Dead 37/112 (33.0%); 14 underlying disease; 12 infection; 3 MOF; 3 GVHD; 1 PTLD; 1 NN; 3 PRES

Supplementary Material 1. Demographic and clinical characteristic of PRES patients from the database

Abbreviation: PRES, posterior reversible encephalopathy syndrome; M, Male; F, Female; **Primary diagnosis:** B-ALL, B cell acute lymphoblastic leukemia; T-ALL, T cell acute lymphoblastic leukemia; AML, acute myelocytic leukemia; HPS, hemophagocytic syndrome; Non-MD, non-malignant disease; NHL, non-hodgkin lymphoma; HL, hodgkin lymphoma; SL, solid tumor; Tha, Thalassemia; AA, aplastic anemia; BL, burkitt's lymphoma; DLBCL, diffuse large B-cell lymphoma; ES, ewing sarcoma; CML, chronic myelogenus leukemia; MDS, myelodysplastic syndrome; FA, Fanconi; HB, hepatoblastoma; WT, wilms'tumor = Hephroblastoma; CPRCA, congenital pure red cell aplasia; CT, congenital thrombocytopenia; AMBL, acute megakaryoblastic leukemia; CDA, congenital dyserythropoietic anemia; NB, neuroblastoma; PNET, primitive neuroectoder tumor. **Oncological Treatment at PRES onset**: CTX, cylocphosphamide; Arc-C, cytarabine; IT, intrathecal injection(Dex+ Arc-C+MTX); Dex, dexamethasone; VCR, vincristine; VDS, vindesine; Ifo, ifosfamide; DNR, daunorubicin; MTX, methotrexate, HD-MTX, high dosage methotrexate; L-Asp, L-Asparaginase; P-Asp, PEG-Asp, pegaspargase; Pre, prednisolone; THP, pirarubicin; VDLD, (Pre+VCR+Dex+L-Asp+DNR+IT); reinductionVDLD, (Pre+VCR+Dex+L-Asp+THP+IT) ; VP16, etoposid; CsA, cyclosporin A; MP, methylprednisolone; 6MP, 6-mercaptopurine; Dox, Doxorubicin; Rit, rituximab; HSCT, hematopoeitic stem cell transpantation; Bu, busulfan; Flu, fludarabine; Tac, tacrolimus; Mmf, mycophenolate mofetil; Tre, treosulfan; Car, carboplatin; Sor, sorafenil; Clo, clofarabine; VM26, teniposide; ATG, anti-thymocyte globulin; TBI, total body irradiation; TT, thiotepa; Tre, treosulfan; Mel, melphalan; **EEG findings** : EEG, electroencephalogram; FS, focal slowing; DS, diffuse slowing; PLEPD, periodic lateralize epileptiform discharge; Oc, occipital lobe; Pa; parietal lobe; Te, temporal lobe ;Fr, frontal lobe ; **Symptoms/Signs** : Sei, seizures; Vis, visual change; Hea, headache; Men,

mental impairment; Vom, vomiting; Hyp, hypertention; **PRES treatment**: *Anti-epileptic drugs*; Dia, diazepam; PB, phenobarbital; Mid, midazolam; VPA, sodium valproate; PHT, phenytoin; Lev, levetiracetam; Clz, clobazam; Lor, lorazepam; *Anti-hypertention* ; ACE-I, angiotensin-converting enzyme inhibitor; CCB, calcium channel blocker; ARB, angiotensin receptor blockor; Nif, nifedipine; Snp, sodium nitroprusside; Diu, Diuretics; Am, amlodipine; Nit, nimodipine; Oxi, oxiracetam; Cap, captopril; Lab, labetalol; Ena, enalapril; Isr, isradipine; Pro, propranolol; Dih, dihydralazine; Clo, clonidine; Met, metoprolol; Ler, lercanidipine; Ram, ramipril; Per, perindopril; **Neuroimaging related:** MRI, magnetic resonance imaging; CT, computerized tomography; *Initial lesion sites* : Oc, occipital lobe; Pa, parietal lobe; Fr, frontal lobe; Te, temporal lobe; Wm, white matter; Hi, hippocampus; CB, cerebellum; CC, corpus callosum; BG, basal ganglia; SC, subcortical; PV, periventricular; Th, thalamus; AC, anterior cortex; PC; posterior cortex; **Follow-up findings:** D, day; M, month; Y, year; CR, complete resolution; PR, partial resolution; RL, residual lesion; NA, not application.

References

1. Eroglu N, Bahadir A, Erduran E. A Case of ALL Developing Posterior Reversible Encephalopathy Secondary to Hyponatremia. *J Pediatr Hematol Oncol* (2017) 39(8), e476-e8. doi: 10.1097/mph.0000000000000827.
2. Cifci Sunamak E, Ozdemir N, Celkan T. Posterior reversible encephalopathy syndrome in children with acute lymphoblastic leukemia: Experience of a single center using BFM protocols. *Pediatric Blood Cancer* (2019) 66(6), e27711. doi: 10.1002/pbc.27711.
3. Kapoor R, Simalti A, Kumar R, Yanamandra U, Das S, Singh J, et al. PRES in Pediatric HSCT: A Single-Center Experience. *J Pediatr Hematol Oncol* (2018) 00(00).
4. Khan SJ, Arshad AA, Fayyaz MB, Ud Din Mirza I. Posterior Reversible Encephalopathy Syndrome in Pediatric Cancer Clinicaland Radiologic Findings. *jgo.org JGO – Journal of Global Oncology* (2017).
5. Fraint E, Miller R, Walter A. Posterior Reversible Encephalopathy Syndrome and Cerebral Sinus Thrombosis in a Case of Pediatric B-Cell ALL. *J Pediatr Hematol Oncol* (2017) 39(2), e71-e3. doi: 10.1097/mph.0000000000000728.
6. Tavil B, Isgandarova F, Bayhan T, Unal S, Kuskonmaz B, Gumruk F, et al. Sorafenib-induced Posterior Reversible Encephalopathy Syndrome in a Child With FLT3-ITD-positive Acute Myeloid Leukemia. *J Pediatr Hematol Oncol* (2016) 38(3), 240-2. doi: 10.1097/mph.0000000000000521.
7. Veljanovska AP, Stojanoski Z, Chadievski L, Stavridis IP, Trajkova S, Cevreska L, et al. Posterior Reversible Encephalopathy Syndrome (PRES) in Children Undergoing Allogeneic Stem Cell Transplantation. *Prilozi (Makedonska akademija na naukite i umetnostite. Oddelenie za medicinski nauki)* (2019) 40(1), 81-6. doi: 10.2478/prilozi-2019-0007.
8. Pavlidou E, Pavlou E, Anastasiou A, Pana Z, Tsotoulidou V, Kinali M, et al. Posterior reversible encephalopathy syndrome after intrathecal methotrexate infusion: a case report and literature update. *Quant Imaging Med Surg* (2016) 6(5), 605-11. doi: 10.21037/qims.2016.10.07.

9. Aureli V, Giammattei L, Maduri R, Daniel RT, Messerer M. Posterior reversible encephalopathy syndrome (PRES) due to neuroblastoma in a child presenting with acute hydrocephalus. *Childs Nerv Syst* (2018) 34(1), 15-7. doi: 10.1007/s00381-017-3640-6.
10. Musiol K, Waz S, Boron M, Kwiatek M, Machnikowska-Sokolowska M, Gruszczynska K, et al. PRES in the course of hemato-oncological treatment in children. *Childs Nerv Syst* (2018) 34(4), 691-9. doi: 10.1007/s00381-017-3664-y.
11. Shkalim-Zemer V, Konen O, Levinsky Y, Michaeli O, Yahel A, Krauss A, et al. Calcineurin inhibitor-free strategies for prophylaxis and treatment of GVHD in children with posterior reversible encephalopathy syndrome after stem cell transplantation. *Pediatr Blood Cancer* (2017) 64(11). doi: 10.1002/pbc.26531.
12. Danhofer P, Tomeckova M, Cerna D, Zapletalova D, Horak O, Aulicka S, et al. Prognostic factors and seizure outcome in posterior reversible encephalopathy syndrome (PRES) in children with hematological malignancies and bone marrow failure: A retrospective monocentric study. *Seizure* (2019) 72, 1-10. doi: 10.1016/j.seizure.2019.08.007.
13. Tambasco N, Mastrodicasa E, Salvatori C, Mancini G, Romoli M, Caniglia M, et al. Prognostic factors in children with PRES and hematologic diseases. *Acta Neurol Scand* (2016) 134(6), 474-83. doi: 10.1111/ane.12570.
14. Hafez HA, Ragab I, Sedky M, Shams M, Youssef A, Refaat A, et al. Patterns, risk factors and outcome predictors of posterior reversible encephalopathy syndrome in pediatric cancer patients. *Leukemia and Lymphoma* (2020). doi: 10.1080/10428194.2020.1832658.
15. Lin W, Xie J, Zhang J, Cheng H, Cui H, Zhang Y, et al. Posterior reversible encephalopathy syndrome in children with acute lymphoblastic leukemia during remission induction chemotherapy: a single-center retrospective study. *Minerva pediatrica* (2019). doi: 10.23736/S0026-4946.19.05675-5.
16. Li XY, Huang K, Zhou DH, Li Y, Xu HG, Weng WJ, et al. Severe hypertension is an independent risk factor for posterior reversible encephalopathy syndrome post-hematopoietic cell transplantation in children with thalassemia major. *Clin Transplant* (2019) 33(1), e13459. doi: 10.1111/ctr.13459.
17. Anastasopoulou S, Eriksson MA, Heyman M, Wang C, Niinimaki R, Mikkel S, et al. Posterior reversible encephalopathy syndrome in children with acute lymphoblastic leukemia: Clinical characteristics, risk factors, course, and outcome of disease. *Pediatr Blood Cancer* (2019) 66(5), e27594. doi: 10.1002/pbc.27594.
18. Khan RB, Sadighi ZS, Zabrowski J, Gajjar A, Jeha S. Imaging Patterns and Outcome of Posterior Reversible Encephalopathy Syndrome During Childhood Cancer Treatment. *Pediatr Blood Cancer* (2016) 63(3), 523-6. doi: 10.1002/pbc.25790.
19. Gaziev J, Marziali S, Paciaroni K, Isgro A, Di Giuliano F, Rossi G, et al. Posterior Reversible Encephalopathy Syndrome after Hematopoietic Cell Transplantation in Children with Hemoglobinopathies. *Biol Blood Marrow Transplant* (2017) 23(9), 1531-40. doi: 10.1016/j.bbmt.2017.05.033.
20. Chen Q, Zhao X, Fu HX, Chen YH, Zhang YY, Wang JZ, et al. Posterior reversible encephalopathy syndrome (PRES) after haploidentical haematopoietic stem cell transplantation: incidence, risk factors and outcomes. *Bone Marrow Transplant* (2020) 55(10), 2035-42. doi: 10.1038/s41409-020-0894-5.

21. Zama D, Gasperini P, Berger M, Petris M, De Pasquale MD, Cesaro S, et al. A survey on hematology-oncology pediatric AIEOP centres: The challenge of posterior reversible encephalopathy syndrome. *Eur J Haematol* (2018) 100(1), 75-82. doi: 10.1111/ejh.12984.
