# Supplementary figures and images for: Management and Clinical Outcome of Posterior Reversible Encephalopathy Syndrome in Pediatric Oncologic/Hematologic Diseases: A PRES Subgroup Analysis With a Large Sample Size
Source: Front Pediatr. 2021 Jul 1;9:678890. doi: 10.3389/fped.2021.678890 (PMC8280768; doi:10.3389/fped.2021.678890)

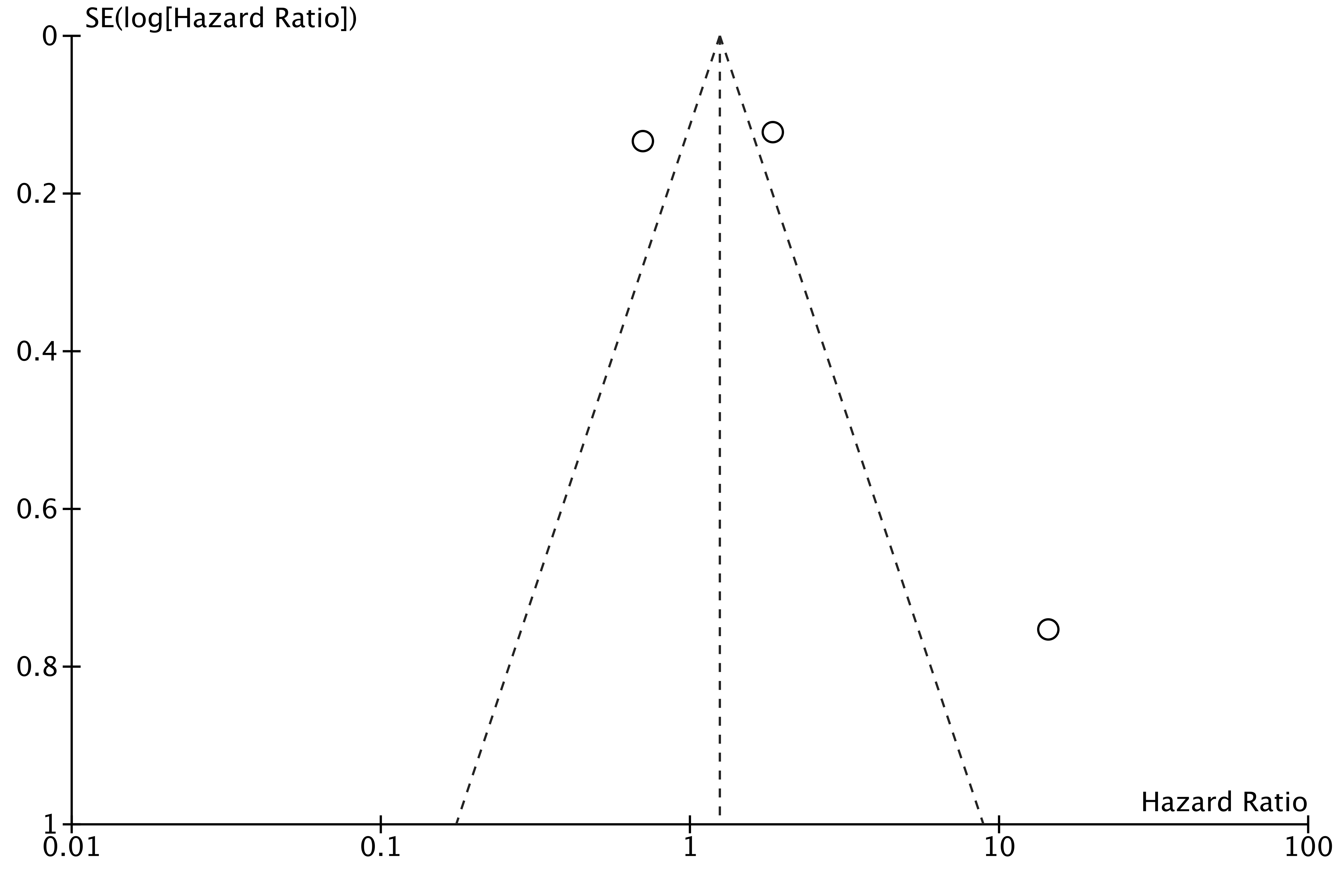

Supplement: Supplementary file 2 [file Image_1.TIF]
